# ggVennDiagram: An Intuitive, Easy-to-Use, and Highly Customizable R Package to Generate Venn Diagram

**DOI:** 10.3389/fgene.2021.706907

**Published:** 2021-09-07

**Authors:** Chun-Hui Gao, Guangchuang Yu, Peng Cai

**Affiliations:** ^1^State Key Laboratory of Agricultural Microbiology, State Environmental Protection Key Laboratory of Soil Health and Green Remediation, College of Resources and Environment, Huazhong Agricultural University, Wuhan, China; ^2^Department of Bioinformatics, School of Basic Medical Sciences, Southern Medical University, Guangzhou, China

**Keywords:** Venn diagram, grammar of graphic, data visualization, R software, ggplot2

## Abstract

Venn diagrams are widely used diagrams to show the set relationships in biomedical studies. In this study, we developed ggVennDiagram, an R package that could automatically generate high-quality Venn diagrams with two to seven sets. The ggVennDiagram is built based on ggplot2, and it integrates the advantages of existing packages, such as venn, RVenn, VennDiagram, and sf. Satisfactory results can be obtained with minimal configurations. Furthermore, we designed comprehensive objects to store the entire data of the Venn diagram, which allowed free access to both intersection values and Venn plot sub-elements, such as set label/edge and region label/filling. Therefore, high customization of every Venn plot sub-element can be fulfilled without increasing the cost of learning when the user is familiar with ggplot2 methods. To date, ggVennDiagram has been cited in more than 10 publications, and its source code repository has been starred by more than 140 GitHub users, suggesting a great potential in applications. The package is an open-source software released under the GPL-3 license, and it is freely available through CRAN (https://cran.r-project.org/package=ggVennDiagram).

## Introduction

A Venn diagram is a widely used diagram that shows the relationships between multiple sets. In biomedical studies, a Venn diagram is frequently used in distinguishing the membership of various types of data, such as compounds, genes, pathways, and species. When the number of sets is less than five, Venn diagrams are probably the most intuitive form of data visualization, superior to heat maps and tables.

In the R environment, one of the most popular platforms in biomedical data visualizations, many packages are available to plot a Venn diagram including VennDiagram (Chen and Boutros, 2011), colorfulVennPlot (Noma and Manvae, 2013), venn (Dusa, 2020), nVennR (Quesada, 2021), eulerr (Larsson, 2020), venneuler (Wilkinson, 2011), RVenn (Akyol, 2019), and gplots (Warnes et al., 2020), to name a few (see Table 1 for a feature comparison of these packages). As one of the most popular software, VennDiagram supports multiple input formats, and it can also generate Euler diagrams in addition to Venn. In addition, venn supports the drawing of Venn diagrams with up to seven sets. RVenn has been developed as a systematic and easy-to-use method for calculating intersecting and overlapping members in Venn diagrams. It is impossible to develop a state-of-the-art Venn tool without absorbing the strengths of the above-mentioned tools.

**TABLE 1:** Feature comparisons of currently available Venn plot tools (R packages and web tools).

	**Grammar of graphics**	**Data processing**			**Visualization**				**References**
		**Access to region members**	**Input format**	**Structured data storage**	**Region filling^*a*^**	**Shapes**	**No. of sets**	**Element control (set and region)**	
**R packages**									
ggVennDiagram	Fully support	Yes	List	Yes	Yes	Circle, ellipse, and others	2–7	Set edge/label, region filling/label	Gao, 2021
VennDiagram	No	Yes	List	No	No	Circle, ellipse	2–5	Set edge/label/filling/area, region label	Chen and Boutros, 2011
colorfulVennPlot	No	No	Named vector	No	Yes	Circle, ellipse	2–4	Set label, region filling/label	Noma and Manvae, 2013
venn	No^*b*^	No	List, formula, set number, Boolean values	No	Yes	Circle, ellipse, and others^*c*^	2–7^*c*^	Set edge/label, region filling/label	Dusa, 2020
nVennR	Partial	Yes	List	Yes	No	Irregular polygon (calculated)	2–many	Set edge/filling/area, region label	Quesada, 2021
eulerr	No	No	List, data frame, table, matrix, named vector	No	No	Circle, ellipse	2–4, maybe many^*d*^	Set label/filling/area, region label	Larsson, 2020
venneuler	No	No	Formula, matrix, character vector	No	No	Circle	2–4, maybe many^*d*^	Set label/filling/area	Wilkinson, 2011
RVenn	No	Yes^*e*^	Venn object (derived from list)	No	No	Circle	2–3	Set filling/edge	Akyol, 2019
gplots	No	Yes	List, data frame	No	No	Circle, ellipse	2–5	Set label, region label	Warnes et al., 2020
**Online webtool**									
InteractiVenn	na	Yes	List (web interface)	na	No	Circle, ellipse, and Edwards	2–6	Set label/filling, region label	Heberle et al., 2015
Venny	na	Yes	List (web interface)	na	Yes	Circle, ellipse	2–4	Set label, region label/filling	Oliveros, 2007

*^a^Region filling indicates that every single part of set intersections/overlapping can be specified separately.*

*^b^venn has a parameter (“ggplot”) to enable the output of a *ggplot* object in plotting.*

*^c^The five- to seven-set Venn diagram is plotted by ggVennDiagram on the basis of venn.*

*^d^When the relationship of different sets is simple enough, eulerr and venneuler can produce an area-proportional Euler plot with more than four sets.*

*^e^Set operation of RVenn is expanded in ggVennDiagram to calculate the shapes in different regions. na, not applicable.*

However, the above-mentioned software packages also have their disadvantages. First of all, these packages have limitations in displaying the difference between various regions in a Venn diagram in spite of the capability of exhibiting the original sets. ColorfulVennPlot and venn do support region filling, but users need to manually specify colors for every region, making it too complicated to be used by ordinary users. Besides, most of these packages lack full support for grammar of graphics, resulting in the failure of adequate integration into the popular ggplot2 ecosystem. In addition, the inputs of some packages are very obscure; thus, it is time-consuming to obtain a qualified input data.

Considering this, we developed ggVennDiagram, an intuitive, easy-to-use, and customizable R package to generate Venn diagrams, which supports a two- to seven-set Venn plot and generates publication-quality figure with minimal input. Furthermore, we also developed a comprehensive Venn data structure to simplify the expansion of Venn diagrams and make the new presentation of the diagram easy in the future.

## Results and Discussion

### Workflow of ggVennDiagram

The main function “ggVennDiagram()” accepts a list input and outputs a *ggplot* object. By measuring the length of input list, it automatically applies internal functions to build a plot in two steps: data pre-processing and visualization. The second step relies on ggplot2’s functions; therefore, we mainly focus on explaining the first step as follows.

Data pre-processing then can be divided into two procedures: shape generation, which defines the edges of Venn sets and regions and region value calculation which calculates the region items and performs necessary statistics, such as counting and calculating percentages.

**FIGURE 1:**
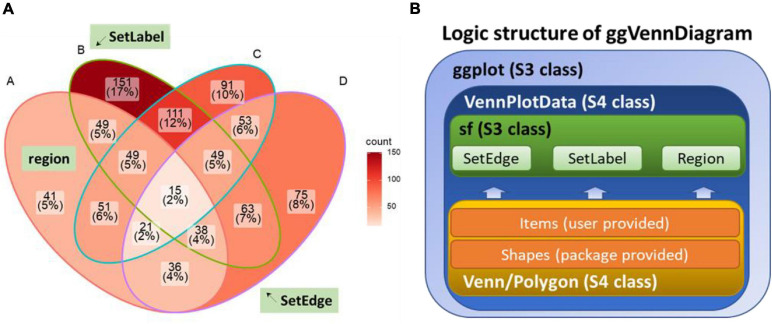
Design of ggVennDiagram. **(A)** Components of a Venn diagram. *SetEdge*, *SetLabel*, and *region* are highlighted with a green textbox. **(B)** Logic structure of ggVennDiagram. The Venn diagram plotted by this package is a *ggplot* object that stores *VennPlotData* object. The *VennPlotData* object is further compiled by the simple features described in sf and the *Venn*/*Polygon* object introduced from RVenn.

Since the returned data after data pre-processing are compatible with the *sf* object, these data are directly passed into “geom_sf()”/“geom_sf_label()”/“geom_sf_text()” functions intrinsically provided by ggplot2. Filling colors are mapped to the counts of region items, and a color bar legend is generated automatically to show the difference between different regions (Figure 1A).

### Shape Generation

In ggVennDiagram, we treated all the edges, labels, and polygons as simple features, which refer to a standard to describe how the objects in the real world can be presented in computers, with emphasis on the spatial geometry of these objects. A total of 15 types of simple features are implemented in R, three of which are used to describe all the components of a Venn diagram.

Firstly, the edges of sets are inherited from *LINESTRING*, which is a sequence of points connected by straight non-self-intersecting lines. Secondly, all the possible intersecting regions are inherited from *POLYGON*, which is formed by a sequence of closed points. Thirdly, the labels of sets are inherited from *POINT*, which is a single point used to anchor a short text. Simple features are to define the coordinates of Venn plot components. It is the first time for simple features to be employed in a Venn diagram. Such a design enhances the ability to describe Venn diagram components, making it possible to calculate intersection and overlapping regions between different sets.

To simplify the calculation of simple features, we introduce an S4 class *Polygon* object which expands the S4 class *Venn* object derived from RVenn. As those methods are implemented in RVenn, set operation methods are implemented for *Polygon* object, resulting in the unified set operation functions for the set object *Venn* and the shape object *Polygon*.

The shape used in the Venn diagram with less than four sets can be a simple structure, such as a circle or an ellipse, but when the Venn diagram has more than four sets, irregular polygons are required. It is hard to generate irregular polygons with simple geometric functions. Therefore, ggVennDiagram is designed to bear a built-in preprocessed shape data set imported from venn, VennDiagram, and some online materials, which undoubtedly increases the efficiency of shape generation on the user side.

### Region Value Calculation

Region value calculation depends on the RVenn package and new functions written on its defined *Venn* object. There are a total of 2^n^ - 1 regions in a Venn diagram, in which n indicates the number of sets. The member and its number in each region are stored with region IDs in a *tibble* and joined with the region shape object through unique IDs. Likewise, the member and its number in a set are assigned to the *SetEdge* through unique IDs in parallel. By doing this, a complete *VennPlotData* object is generated for subsequent plotting (Figure1B).

### Stepwise Self-Customization of Venn Diagrams

**FIGURE 2:**
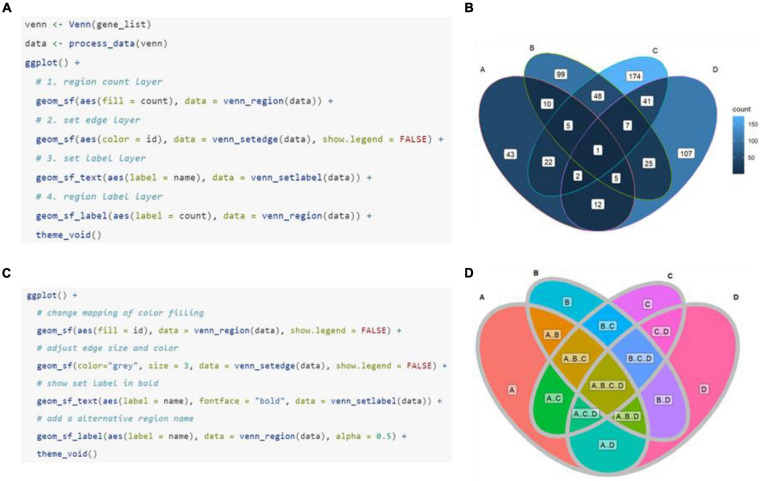
Plotting method of ggVennDiagram. The default manner **(A)** and returned plot **(B)** when user calls “ggVennDiagram()” with a four-set list of genes (“gene_list”). **(C,D)** Stepwise self-customization of the Venn plot by using ordinary ggplot2 functions.

After data pre-processing, ggVennDiagram calls native ggplot2 functions to draw Venn diagrams in four layers (Figures 2A,B). The first layer is to show the number of members in each region, with gradient color filling exhibiting the differences in member number among various regions. The second layer is to show set edges. When an irregular polygon rather than an ellipse and circle is used to draw a Venn diagram, set edges are essential for distinguishing the boundary between different sets. The third layer is to display set labels, and the fourth layer is to exhibit region labels. The data pre-processing function is accessible to users. Thus, it is easy for those familiar with the ggplot2 syntax to revise the details of the image including the region fill color, line color/thickness, text style, and so on (Figures 2C,D).

### Novel Shapes in Venn Diagrams

**FIGURE 3:**
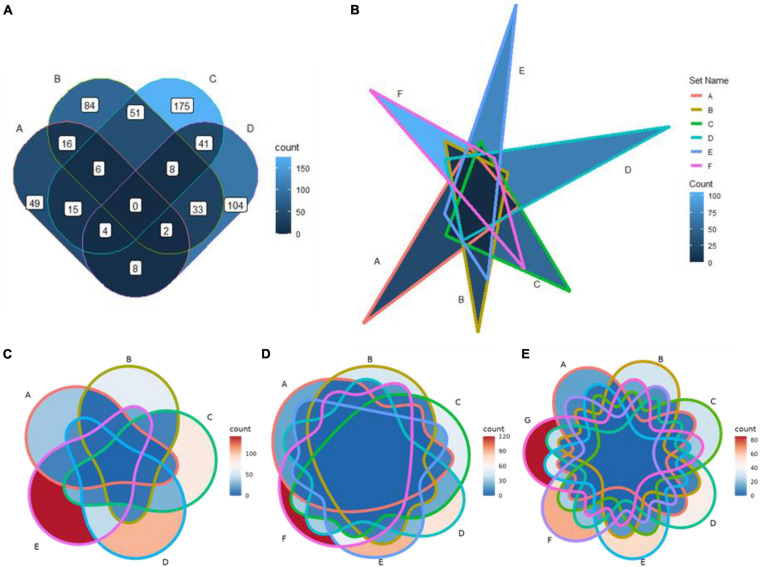
Application of new shapes and support for a Venn diagram of up to seven sets in ggVennDiagram. **(A)** A rounded rectangle is used to plot a four-set Venn diagram. **(B)** A triangle is used to plot a six-set Venn diagram. **(C–E)** Five-, six-, and seven-set Venn diagrams plotted based on ggVennDiagram. The reproducible examples are available from the vignettes attached to the package.

As has been noted above, a set of built-in shapes from ggVennDiagram is used to plot the Venn diagram. By default, only the most appropriate shape is used when the main function “ggVennDiagram()” is called. However, other applicable shapes can be specified in a stepwise plot, which has been described in the previous section (Figure 3A). In addition, ggVennDiagram provides a series of functions to help users with a novel shape when they know shape coordinates. For example, a six-set Venn diagram can be made up of only six triangles (Figure 3B). To this end, we just need to pass the vertex coordinates and set label coordinates to the “triangle()” function and “label_position()” function, respectively, and then construct a *VennPlotData* object with the constructor function “VennPlotData()” (Figure 1B). The generated *VennPlotData* object now can join with set and calculated region values through “plotData_add_venn()” function, and the resultant data can be used in stepwise customization of the Venn diagram (Figure 3B).

### Venn Diagram With More Than Four Sets

From version 1.0, ggVennDiagram supports Venn diagrams with up to seven sets (Figures 3C–E). This feature is dependent on the shapes imported from another R package venn (Dusa, 2020). However, we insist that Venn diagrams with more than four sets may not be a good choice to display their relationships.

To date, there are three major methods to display set relationships: Venn diagram, Euler diagram, and UpSet plot (Conway et al., 2017). The UpSet plot is a state-of-the-art visualization technique for the quantitative analysis of sets (Lex et al., 2014), and it supports an unlimited number of sets. When the number of sets is very large, it is more justified to choose the UpSet plot.

### Integration of ggVennDiagram Into Bioinformatics Analysis Pipelines

The first version of ggVennDiagram was released on October 9th, 2019 (version 0.3). Since then, it has been applied to many biomedical research fields. For example, Cook et al. (2020) used ggVennDiagram to show overlapping differentially expressed genes across three sample times (days 1, 3, and 5) in both the root and the shoot of canola. Besides, Harris et al. (2020) used ggVennDiagram to display that 22.5% of differentially expressed genes were shared by treated mice and human patients. Furthermore, Maguire et al. (2020) used ggVennDiagram to confirm that their novel method has low bias and is more sensitive than three other methods for small RNA library preparation. In addition, ggVennDiagram is also used for analyzing the differences between several spatially varied oral metabolomics samples (Ciurli et al., 2021) and for comparing single-nucleotide variants between tumor and non-tumor tissues (Horny et al., 2021). So far, ggVennDiagram has been cited in more than 20 peer-reviewed articles and open-access preprints, as retrieved by Google Scholar. It could be speculated that ggVennDiagram has a very wide range of application scenarios in biomedical studies.

### Feature Comparisons of Currently Available Venn Plot Tools

**FIGURE 4:**
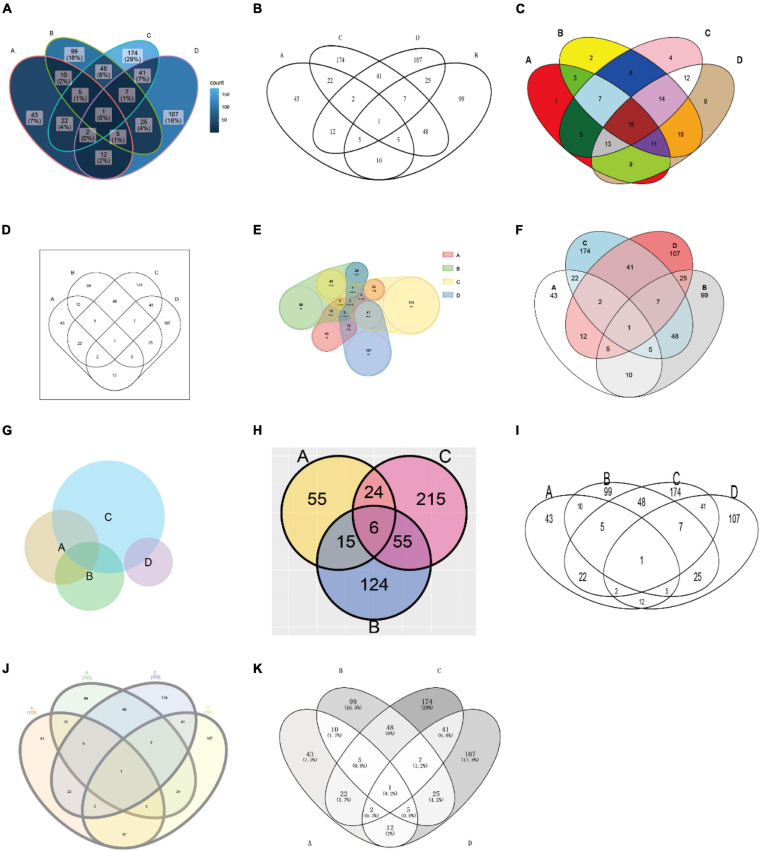
Plots generated by the tools listed in Table 1. The used tools are ggVennDiagram, VennDiagram, colorfulVennPlot, venn, nVennR, eulerr, RVenn, gplots, interactiVenn, and Venny **(A–K)**, respectively. Except for venn **(D)**, venneuler **(G)**, and RVenn **(H)**, all plots are generated with minimal configuration (using default parameters) and the same input data, which is a simulated four-set gene list. The input for venn is a named vector, while the input for venneuler is a qualified expression. Since RVenn do not support four-set Venn plots, only three sets of the gene list are used **(H)**.

Table 1 presents the features of currently available Venn plot tools (see also Figure 4 for the comparison of the generated plots by these tools). First of all, the support for grammar of graphics by nine R packages and two web tools was assessed. Grammar of graphics is a general scheme for data visualization, which breaks up graphs into semantic components, such as scales and layers. Except ggVennDiagram, none of these tools fully support this feature in plotting Venn diagrams.

Additionally, ggVennDiagram takes the lead in the following three aspects of data processing capacity. (1) We can get access to region members by querying the *VennPlotData* object. (2) It should be noted that we only implement the input of list (as input format). This design is simple enough to understand and prepare, and it is easy to store set members, which is essential for the calculation of region members. (3) *Via* the design of a layered object, ggVennDiagram can store plotting data into the *VennPlotData* object (Figure 1B), thus making it possible to query and reuse the target data.

Furthermore, ggVennDiagram is superior in four aspects of visualization. (1) Region filling allows the user to easily identify the differences between various parts of the Venn diagram, and this is one of the key features of ggVennDiagram. Although several other tools have this feature, only ggVennDiagram is fully automatic since it is driven by ggplot2’s aesthetic mapping. (2) The ggVennDiagram has built-in shapes consisting of circles, ellipses, and others. Besides, we also provide functions to help users to import self-defined shapes (Figures 3A,B). (3) The ggVennDiagram supports two- to seven-set Venn diagrams, which is adequate for daily use. (4) Element control in ggVennDiagram can be applied for set edge/label and region filling/label, so that it is convenient to set their color/line type/size, and so on (Figures 1A, 2B,D,3A–E).

Notably, several tools support both Venn and Euler diagrams. However, an Euler diagram has two shortages: firstly, it is area proportional, but the human eye is less sensitive to area than to color; secondly, it only shows relevant relationships, but sometimes, it is impossible to show all intersection regions merely by using simple geometric shapes, such as circles and ellipses. Therefore, we assume that it is more appropriate to use color filling for displaying the difference between different regions in ordinary biomedical studies.

Overall, ggVennDiagram integrates and optimizes a Venn diagram plotting method, exhibiting multiple advantages in performance over current existing tools. Compared with webtool, R scripts are easier to integrate into the existing bioinformatics analysis pipelines to realize automation and batch drawing of Venn diagrams. Therefore, it is necessary and useful to develop ggVennDiagram.

## Data Availability Statement

The ggVennDiagram R package is open source and freely available on CRAN (https://cran.r-project.org/package=ggVennDiagram) and GitHub (https://github.com/gaospecial/ggVennDiagram). ggVennDiagram mainly requires R (>3.5.0), the ggplot2, and sf packages, and its full function also depends on the plotly package.

## Author Contributions

C-HG, GY, and PC wrote this manuscript. C-HG implemented this package with the help of GY. PC supervised the project. All authors contributed to the article and approved the submitted version.

## Conflict of Interest

The authors declare that the research was conducted in the absence of any commercial or financial relationships that could be construed as a potential conflict of interest.

## Publisher’s Note

All claims expressed in this article are solely those of the authors and do not necessarily represent those of their affiliated organizations, or those of the publisher, the editors and the reviewers. Any product that may be evaluated in this article, or claim that may be made by its manufacturer, is not guaranteed or endorsed by the publisher.
